# Molecular characterization of the brain secretory peptides, prothoracicotropic hormone (PTTH) and bombyxin, of the silkmoth *Bombyx mori*

**Published:** 2004-05-01

**Authors:** Hironori Ishizaki

**Affiliations:** Professor Emeritus, Nagoya University

**Keywords:** PTTH, bombyxin, brain secretory peptide, *Bombyx mori*, *Samia cynthia ricini*

## Abstract

Molecular characterization of the brain secretory peptides, PTTH and bombyxin, of *Bombyx mori* is reviewed. PTTH is a 30-kDa homodimeric glycoprotein, the monomer of which consists of 109 amino acids. Two monomers are held together by a disulfide bond. cDNA and gene coding for PTTH were cloned and the precursor protein for PTTH monomer was deduced. A novel 5-kD brain secretory peptide named bombyxin has been discovered from *Bombyx* brain. Bombyxin is highly homologous to vertebrate insulin-family peptides and possesses the prothoracicotropic activity when injected into brain-removed pupae of a heterologous moth, *Samia cynthia ricini*, though inactive to *Bombyx* from which it was derived. cDNA and gene coding for bombyxin were cloned, preprobombyxin protein was deduced, and posttranslational processing to generate mature bombyxin was suggested. The *Bombyx* genome contains highly multiple copies of the gene coding for bombyxins. Immunohistochemically, PTTH- and bombyxin-producing brain neurosecretory cells were identified.

## Introduction

The prothoracicotropic hormone (PTTH) secreted from the brain neurosecretory cells turns on the prothoracic glands so as to secrete ecdysone, the steroid which dictates insect cells to develop towards moult and metamorphosis.1) PTTH therefore plays a central role in the control of the insect postembryonic development.

In 1960, soon after entering the graduate school of Kyoto University, I began to purify PTTH from brains of the commercial silkworm *Bombyx mori*. Apparently, *Bombyx* is the most suitable insect as a material for PTTH extraction since a large number of animals are readily available because of the prosperity of sericultural industry in Japan. On the other hand, as the animal to assay PTTH, brain-removed, development-arrested pupae of another moth *Samia cynthia ricini* were used. My supervisor in Kyoto University, late Prof. Mamori Ichikawa (1903~1971), who had studied the biological aspects of PTTH for a long time, advised me to use this assay animal. Importantly, *Samia* brainless pupae were caused to develop when transplanted with *Bombyx* brain, suggesting that *Bombyx* PTTH was species-nonspecifically active to *Samia*. Moreover, he emphasized technical advantages of using *Samia*, compared to *Bombyx*, because *Samia* was easier to rear and the surgery of brain extirpation also easier in this species.

It became soon clear that PTTH is a peptide, and a considerable progress in purification was achieved.2)–4) In 1969, an 8,000-fold purification was achieved to obtain a considerably purified preparation, as little as 0.002 μg of which was able to evoke adult development in a brainless *Samia* pupa.4) The preparation was apparently far from being pure, however, judging from the chromatographic profiles.

A turning point has come in 1970 in the history of my PTTH purification. Prof. Saburo Tamura (now Prof. Emeritus) of the University of Tokyo, Department of Agricultural Chemistry, offered me to collaborate the purification of PTTH. This was a long eagerly waited offer by a biochemist; it had become clearer as purification advanced that this project was too tough for a biologist alone to complete. S. Tamura was a well-known biochemist famous of his pioneering works to characterize a number of novel biologically active substances. Since then, collaboration with Dr. Akinori Suzuki who succeeded Prof. Tamura a few years later, and many young biochemists of Suzuki’s laboratory, among whom Drs. Hiromichi Nagasawa and Hiroshi Kataoka (now Profs. of the University of Tokyo) were the most important contributors to this project, lasted 30 years until PTTH was finally fully characterized. Prof. A. Suzuki (now Prof. Emeritus) has played an excellent leadership throughout this joint research.

We divided labor according to our specialties. All chemical works including purification and chemical analyses of PTTH were done in Tokyo, lyophilized purified fractions were sent to Nagoya, and all biological works including PTTH bioassay was done in Nagoya (I was transferred to Nagoya University in 1973 from Kyoto University). This division of labor brought about a markedly accelerated progress in the joint research.

Meanwhile an astonishing fact was disclosed about 10 years after this joint research started, when PTTH purification seemed to be almost complete. A purified sample was assayed by brainless *Bombyx* pupae to ascertain that the preparation was certainly active to *Bombyx* itself, but contrary to expectation, *Bombyx* brainless pupae did not respond to this sample at all. At an early stage of this research, a crude extract from *Bombyx* brains had been shown to evoke adult development in *Bombyx* brainless pupae, and hence we postulated that the *Bombyx* PTTH was species-nonspecifically active to both *Bombyx* and *Samia*. But now a purified preparation was not so; it was active to *Samia* but not to *Bombyx*. This fact was consistent with the idea that there exist two distinct molecules, one of which is active to only *Samia* while the other is active to only *Bombyx*, and both coexisted in the original, crude extract but were separated from each other by subsequent purification.

Reexamination of each step of the purification scheme so far established readily disclosed that the acetone fractional precipitation, the fourth step of the purification scheme, in fact separated the *Samia*-active molecule from the *Bombyx*-active molecule: a 65~75% acetone precipitate was active when tested with *Samia* while a 40~50% acetone precipitate turned out active with *Bombyx* assay, the latter of which was inactive with *Samia* assay so that must have had been discarded during purification.5) The two molecules also exhibited different behaviors on Sephadex gel-filtration, suggesting that they were different in molecular size; *Samia*-active substance was ~4 kD while *Bombyx*-active substance was ~22 kD. The molecule active with *Samia* assay was named bombyxin6) (called S-PTTH or 4 K-PTTH for some time7)), while that which was active with *Bombyx* assay was termed simply PTTH of *Bombyx* (called B-PTTH or 22 K-PTTH previously).

The genuine PTTH of *Bombyx mori* naturally should be a molecule that is produced by the brain of *Bombyx* and stimulates the prothoracic glands of its own, and therefore the B-PTTH (22 K-PTTH) must be regarded as the true *Bombyx* PTTH. We started therefore purification of the *Bombyx* PTTH, besides that of bombyxin. Bombyxin is evidently an intriguing, physiologically important molecule, though it has now become clear that it is not the true *Bombyx* PTTH, and so purification and elucidation of molecular structure of bombyxin were also continued.

This review article deals with the molecular natures of these two brain secretory peptides of *Bombyx*, PTTH and bombyxin, which were clarified by a long effort over 30 years by the two laboratories of Tokyo and Nagoya, using 30,000,000 *Bombyx* heads in all as material. Similar review articles have appeared.8),9)

## Primary structure of bombyxin

Purification and amino acid sequencing of bombyxin has mainly been led by H. Nagasawa. After a 14-year effort, a final purification scheme consisting of 15 successive procedures has been established.10),11) Bombyxin comprises many molecular forms which could be satisfactorily resolved only by high performance liquid chromatography (HPLC). Bombyxin-I, one of the heterogeneous molecular forms of bombyxin, was first purified to homogeneity as a single, well-defined peak on an HPLC at the terminal step of purification.10) Fifty μg of bombyxin-I was obtained from 650,000 *Bombyx* heads, and 0.1 ng of this pure material was able to evoke adult development when injected into a *Samia* brainless pupa (calculated to be at a concentration of 3 × 10^–11^ M in hemolymph of pupa). Purification fold was 2 × 10^6^. Ecdysone release by a prothoracic gland *in vitro* was enhanced by addition of the pure bombyxin at a concentration of 1 × 10^–11^ M. Subsequently, several molecular forms of bombyxin, bombyxins-IIs, -III,11) -IV12),13) and -V14) were purified to homogeneity. Chromatographic profiles suggested that there remained still more bombyxins to be purified.

**Fig. 1:**
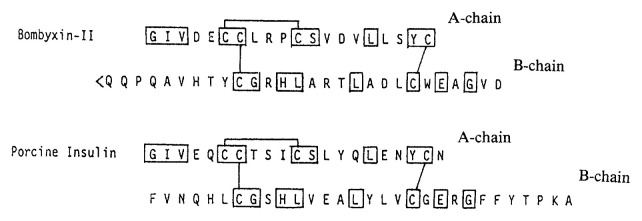
Amino acid sequences of bombyxin-II and porcine insulin. The residues homologous between the 2 peptides are boxed. Upper, A-chain and lower, B-chain. Cys residues forming disulfide bonds are connected by line. < Q denotes pyroglutamate.

The amino-acid sequences were fully determined for Bombyxin-IIs12) and -IV13) and partially for bombyxin-I, -III11) and -V.14) They are the heterodimers consisting of two chains, termed A- and B-chains, which are held together by two disulfide bonds. Interestingly, the amino acid sequences of bombyxins were highly homologous with those of insulin-family peptides11),12) (Fig. 1). Two inter- and one intra-chain disulfide bonds are formed at the same positions as in insulin.12) Molecular modeling for the three dimensional structure of bombyxins showed further that bombyxins resemble insulin in adopting the core structure similar to that of insulin.14)

The finding that the insect brain peptide bombyxin shares a common ancestral molecule with the vertebrate pancreatic peptide insulin is not surprising, in view of the existing knowledge that the peptides originally found in the central nervous system were often found in the gastro-entero-pancreatic system, and *vice versa*.15),16)

## Structure of cDNAs and genes coding for bombyxin

**Fig. 2:**
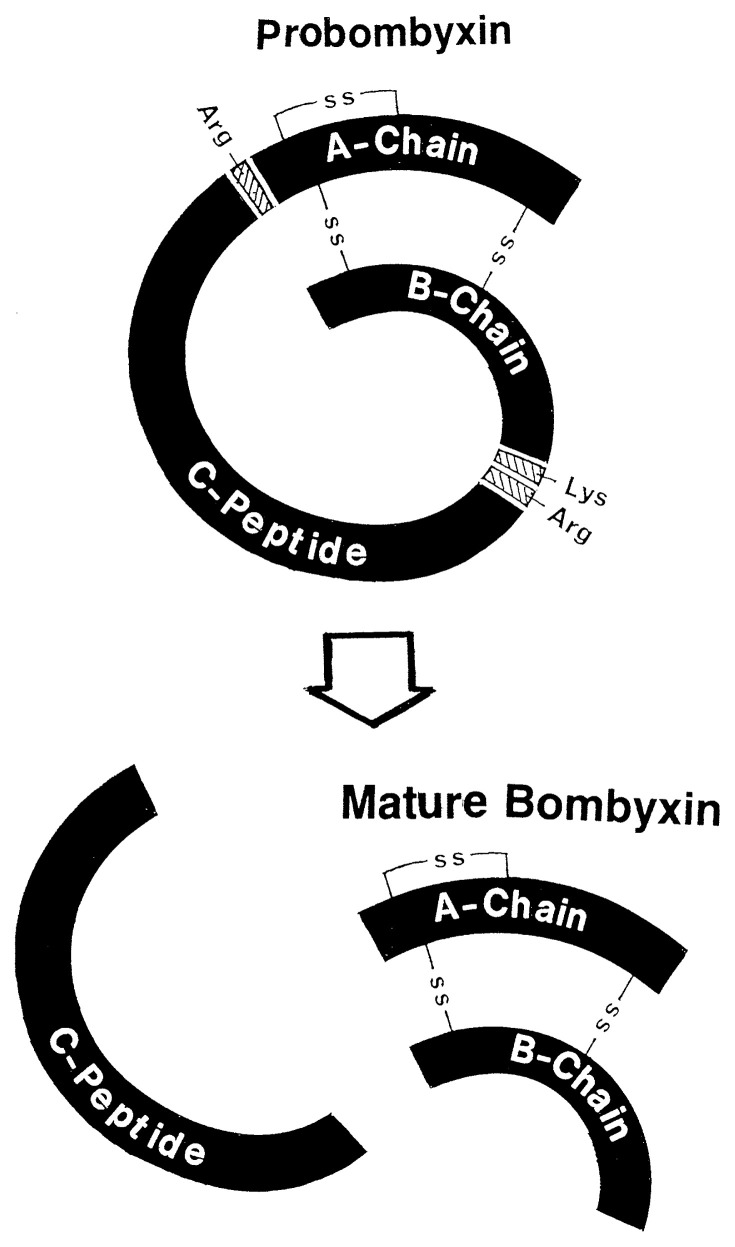
Schematic representation of the formation of the bombyxin mature molecule from probombyxin. C-peptide is presumed to be excised after translation.

By screening a cDNA library prepared from *Bombyx* brain mRNA with a synthetic oligonucleotide probe which was designed based on the amino acid sequence of bombyxin, a cDNA was cloned which encoded the entire length of the presumed precursor protein for bombyxin.17) Thus, the bombyxin cDNA encoded a precursor protein for bombyxin consisting of the signal peptide, B-chain, C-peptide (connecting peptide, comparable to that of proinsulin), and A-chain, in this order from 5’ end. The C-peptide is flanked by a basic and dibasic residues (Arg and Lys-Arg), suggesting that the C-peptide is excised proteolytically after disulfide bond formation between the B- and A-chains, to generate the heterodimeric mature bombyxin (Fig. 2). These structures are precisely the same as those of pre-proinsulin. 18)

**Fig. 3:**
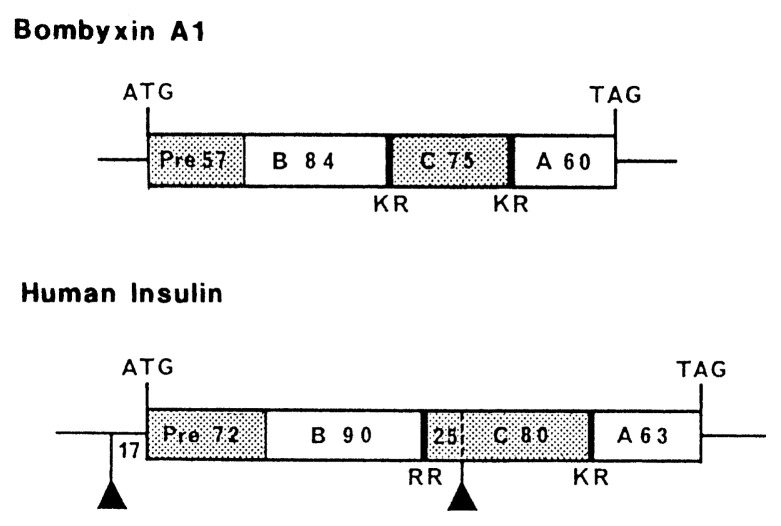
Schematic representation of the genes encoding pre-propeptides for bombyxin A1 and human insulin. Open boxes represent the domains that contribute to the formation of mature peptides. Hatched boxes designate the domains coding for the peptide regions that are excised after translation. Pre, signal peptide; B, B-chain; C, C-peptide; A, A-chain. Numerals indicate the nucleotide numbers. Bold boundaries with KR or RR represent the posttranslational proteolytic cleavage sites. Triangles indicate introns.

Using the same oligonucleotide probe as used for cDNA cloning, a gene for bombyxin was cloned from the *Bombyx* genomic DNA.19) The gene coded for preprobombyxin in exactly the same way as in the bombyxin cDNA (Fig. 3). By using the isolated bombyxin gene as probe, five additional genes were cloned which code for bombyxins with slightly different amino acid sequences.19),20) These data about cDNA and genes substantiated further the notion that bombyxin and insulin share a common ancestral molecule. Drs. Masafumi Iwami, Takashi Adachi and Atsushi Kawakami of Nagoya University mainly contributed to these cDNA and gene studies.

## Highly multiple copies of the bombyxin gene in the *Bombyx* genome

**Fig. 4:**
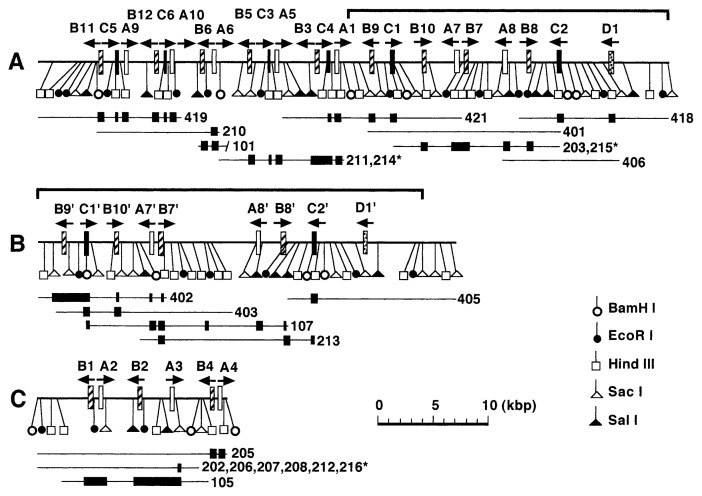
Restriction maps of the three genomic DNA segments (A, B and C) of *Bombyx* (a racial hybrid, Kinshu x Showa). Brackets above maps A and B indicate the regions where the gene and restriction site ditributions are very similar. Segments A and B are thus presumed to be allelic to each other, having derived from each of the parental races Showa and Kinshu. Open, hatched, filled and stippled rectangles on the maps represent the bombyxin family-A, -B, -C and -D genes, respectively. Arrows above the genes indicate their transcriptional directions. Horizontal lines under the maps indicate the overlapping clones. Filled boxes on the lines indicate the sequenced regions.

**Fig. 5:**
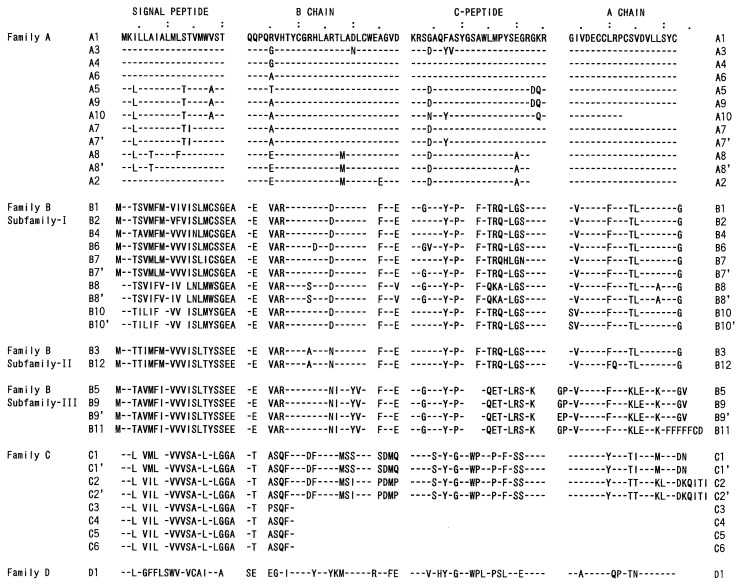
Amino acid sequences of 37 preprobombyxins deduced from the bombyxin genes. They are classified into family A, B, C and D, according to the sequence similarity. Dashes reprent the residues identical with those of preprobombyxin A1. Preprobombyxins with prime (’) are those deduced from the bombyxin genes which are allelic to those which encode corresponding preprobombyxins without prime (Genomic DNA was taken from a racial hybrid, Kinshu x Showa, of *Bombyx*.).

Southern hybridization experiments showed that the *Bombyx* genome contained many copies of bombyxin gene.17),19),20) This was not unexpected in view of the presence of many molecular forms of bombyxin observed during purification.10)–12) Dr. Hidehiko Kondo, a graduate student of Nagoya University at that time (now Head Researcher, Bio-Science Institute, Kao Co. Ltd.) started in 1988 to study in detail this feature of the *Bombyx* genome. By screening a *Bombyx* genomic DNA library with various bombyxin genes so far obtained as probes, 38 genes encoding bombyxins were cloned (Fig. 4).21) Among the 38 genes, 37 genes were sequenced (Fig. 5).21) Five of them were apparently pseudogenes. The 38 bombyxin genes were localized on three genomic DNA segments forming clusters, the largest of which extended over 50-kilo base-pairs (Fig. 4).

**Fig. 6:**
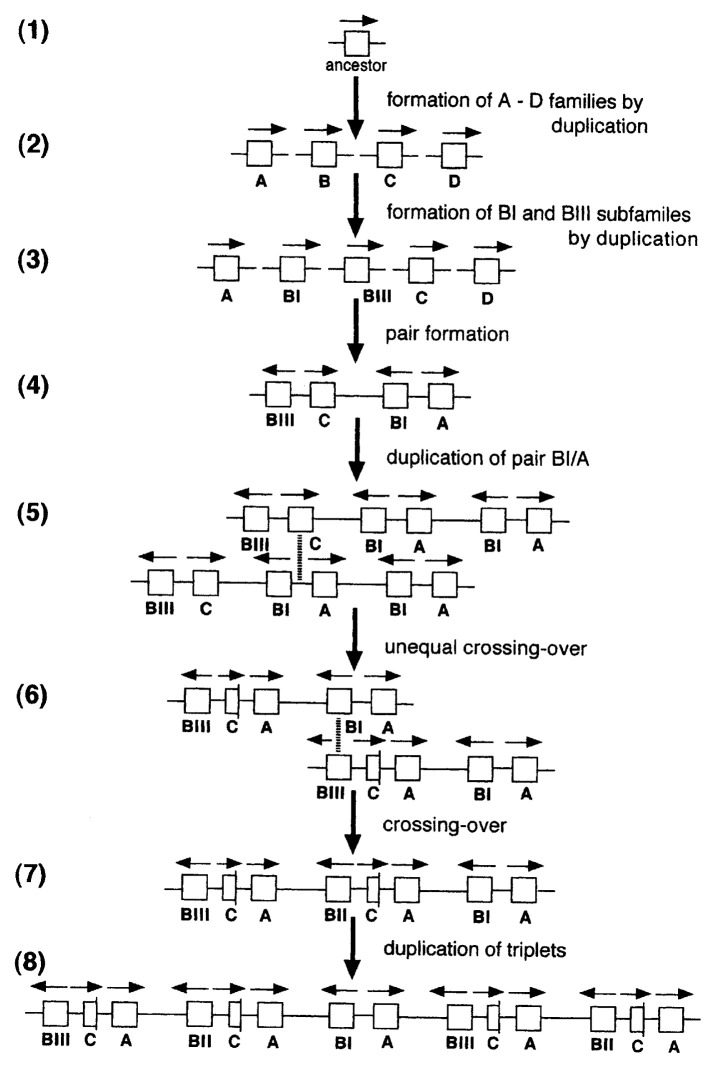
An example showing the evolutionary history of the bombyxin multi-family genes, through mutation, duplication and unequal crossing-over. Arrows above bombyxin genes indicate the transcriptional directions. Vertical broken lines represent points where crossing-over occurred. For further explanation, see Fig. 7 of ref. 21).

The 38 bombyxin genes were classified into A, B, C, and D families according to their similarity in amino acid sequences of deduced preprobombyxins (Fig. 5). All the genes lack introns, contrasting with the vertebrate insulin genes which possess one or two introns.18) These genes are often so arranged as to form pairs or triplets (Fig. 4). In the pairs, two bombyxin genes belonging to the different families are apposed with opposite transcriptional orientation. All triplets are arranged in the order of the family-B, family-C, and family-A genes, and the transcriptional directions of the family-C and family-A genes are opposite to the direction of the family-B gene. Close examination of the intra-gene as well as inter-gene sequences and of restriction maps suggested the occurrence of equal and unequal crossing-over that must have led to the multiplication of bombyxin genes. An example of possible gene rearrangements is schematically shown in Fig. 6.

## Studies using bombyxin antibodies

**Fig. 7:**
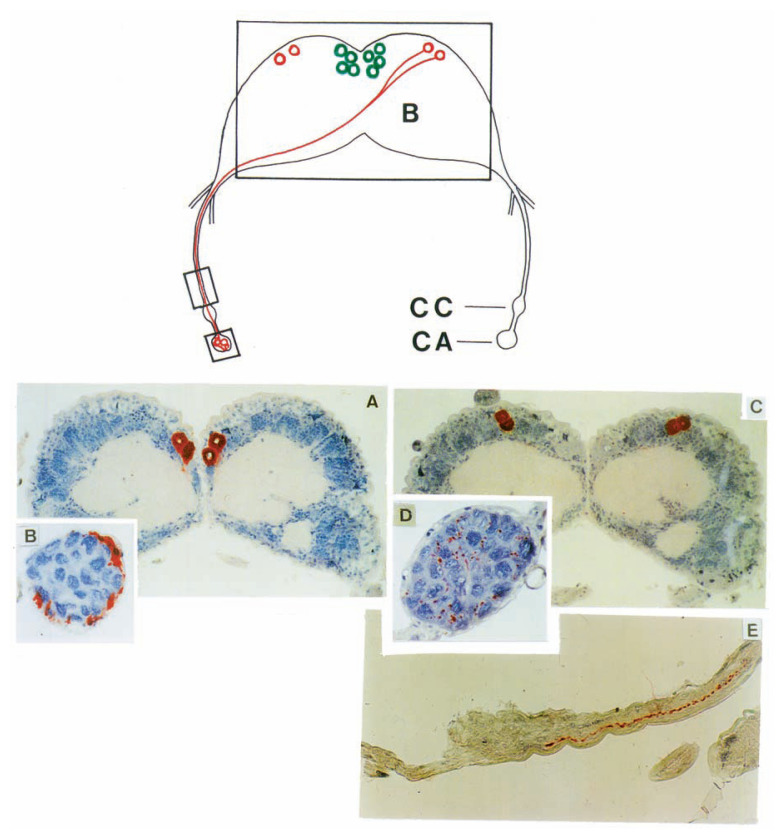
Immunohistochemistry for bombyxin and PTTH in the cephalic endocrine organs of the *Bombyx mori* fifth-instar larva. (Top) Schematic drawing of the brain(B)-corpora cardiaca (CC)-corpora allata (CA)complex. Two pairs of PTTH neurosecretory cells (red) and four pairs of bombyxin neurosecretory cells (green) are present. Only axons emanating from the right PTTH cells to reach the contralateral corpus allatum are shown. (A) Bombyxin cells in the dorsomedial brain. Only two pairs out of four pairs of bombyxin cells are included in this tissue section. (B) Corpus allatum with bombyxin-immunoreactive nerves in the peripheral region. (C) Two pairs of PTTH-immunoreactive dorsolateral brain neurosecretory cells. (D) Corpus allatum with PTTH-immunoreactive nerves deep inside the allatum. (E) Retro-cerebral nerves leading to the corpus cardiacum to show the pathway of PTTH from the neurosecretory cell perikaryon to the corpus allatum. Note the bead-like immunoreactive granules.

Dr. Akira Mizoguchi of Nagoya University has been the leader throughout the studies using the antibodies to bombyxin and PTTH. A peptide corresponding to the N-terminal 10-amino acid sequence of the bombyxin-I A-chain was synthesized and a monoclonal antibody against this synthetic peptide was raised.6) Immunohistochemistry using this antibody revealed the presence of bombyxin in four pairs of large dorsomedial neuro-secretory cells of the *Bombyx* brain (Fig. 7A). The immunoreactive material was traced down to the corpora allata, through the axons that emerge from the neurosecretory cells and cross at central line of brain so that the axons reach the contralateral corpus allatum. The axon endings were localized at the periphery of the allata (Fig. 7B), suggesting that bombyxin is released into blood therefrom.

The monoclonal antibody described above recognized bombyxin by competitive enzyme-linked immunobinding assay only after denaturation of bombyxin by heating or dithiothreitol treatment.6) This antibody was thus useful for immunoblot and immunohistochemical studies, but was not applicable to radio-immunoassay or affinity purification of bombyxin, since the antibody was incapable of recognizing undenatured, bioactive bombyxin. Then, by using a pure preparation of natural bombyxin-II as an antigen, a monoclonal antibody recognizing native bombyxin was prepared.22),23) By radioimmunoassay using this antibody, fluctuation of immunoreactive material in hemolymph during postembryonic development of *Bombyx* was examined.23) The immunoreactivity noticeably increased during pupa-adult development, and the titre in the female was twice as high as the male titre, suggesting the possible role of bombyxin in a sex-associated process(es).

## Primary structure of *Bombyx* PTTH

Work started again on purification of the true *Bombyx* PTTH, a 30-kDa peptide, using *Bombyx* brainless pupae as assay animal. In this work, H. Kataoka was the leading man throughout. After a long-term effort of preliminary purification to search for efficient procedures, a final scheme for PTTH purification was established which consisted of 16 procedures including five different HPLC procedures as the terminal steps.24),25) The increase in the specific activity was as much as 5 × 10^6^ fold, and only 5.4 μg of the purified PTTH was obtained from a batch of 5 × 10^5^ *Bombyx* heads. Adult development in a *Bombyx* brainless pupa was resumed by 0.1 ng of the pure PTTH.

When the intact pure PTTH was subjected to SDS-PAGE a single 30-kDa band was obtained, while SDS-PAGE after reduction by the 2-mercaptoethanol treatment generated two bands of 16–17 kDa. Since these two bands and intact purified PTTH had the same N-terminal amino acid sequence, possessed similar amino acid compositions, and produced almost the same elution pattern when their peptide fragments after proteolytic digestion were subjected to HPLC, it was concluded that PTTH was a dimeric protein composed of two identical or nearly identical subunits which were linked together by a disulfide bond(s). Edman degradation of peptide fragments produced by proteolytic digestion of the purified PTTH elucidated the amino acid sequence of the PTTH subunit up to the 104th residue from the N-terminus. 25) The C-terminal portion remained undetermined, however, because of the limited amount of purified PTTH. The 41st residue was undetermined since no phenylthiohydantoin amino acid was detected at this cycle of degradation.

## cDNA and gene for *Bombyx* PTTH

**Fig. 8:**
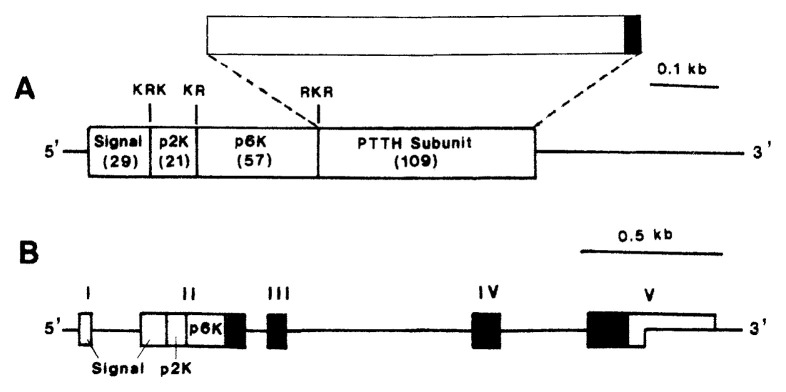
Schematic representation of a cDNA and a gene encoding prepro PTTH-subunit. (A) cDNA. The PTTH subunit (109 amino acids) is encoded by the 3’-end portion of the coding region. The rectangle above the cDNA connected with broken lines to the PTTH-subunit domain of cDNA represents the PTTH-subunit peptide. The open part (104 amino acids) precisely match the amino acid sequence determined by sequencing of purified PTTH, while the solid part (5 amino acids) was newly added by deduction from cDNA.

By screening a cDNA expression library constructed from the *Bombyx* brain mRNA with a mouse polyclonal antibody raised against a synthetic peptide corresponding to the N-terminal 15 amino acids of PTTH subunit, and then with an isolated positive clone as a hybridization probe, seven PTTH cDNA clones were obtained.26) PTTH cDNA encoded a precursor protein for PTTH subunit, consisting of the signal peptide (29 amino acids), two peptide components (21 and 57 amino acids each), and the PTTH subunit (109 amino acids), in this order from the 5’ end (Fig. 8A). Proteolytic cleavage signals bound these components, suggesting that they are separated after translation. The sequence up to the 104th residue of the deduced PTTH subunit, matched the sequence determined by amino acid sequencing of the purified PTTH. It became therefore apparent that the peptide sequencing of the purified PTTH left only five C-terminal residues undetermined. The 41st residue which had not been determined by amino acid sequencing was deduced to be Asn, forming a possible N-linked glycosylation site together with the following two residues (Asn-Lys-Thr).

**Fig. 9:**
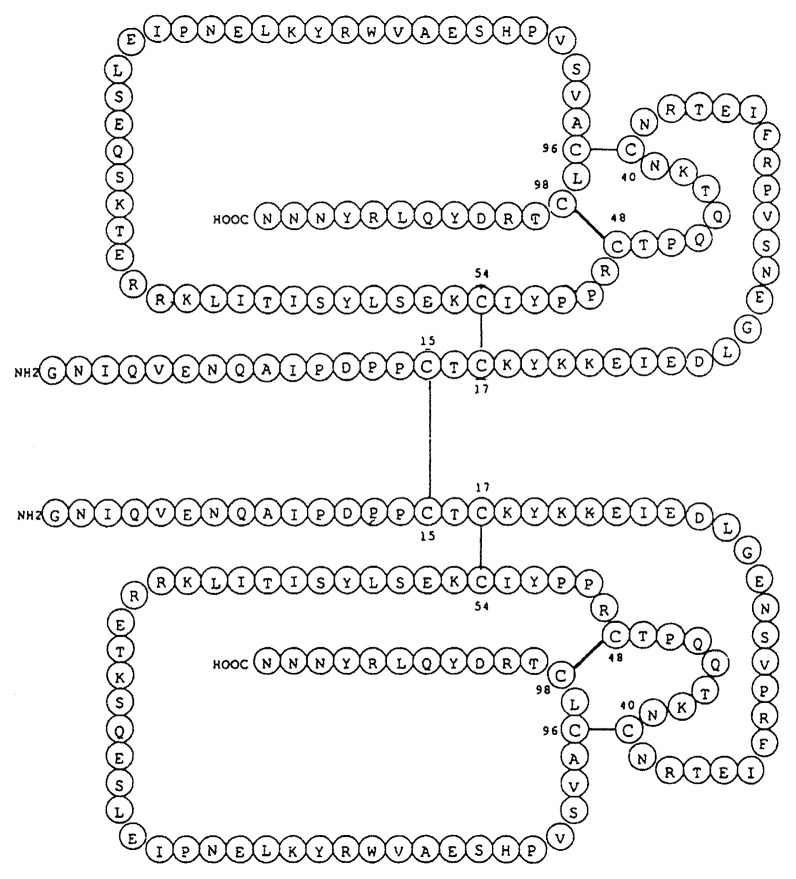
Schematic drawing of the *Bombyx* PTTH primary structure. Parallel orientation of the two subunits is only tentative.

One inter- and three intra-chain disulfide bonds were located using *Escherichia coli* recombinant PTTH.27) The entire structure of a homodimeric mature PTTH thus clarified is schematically shown in Fig. 9. Overall procedures for primary structure determination has been reviewed.28)

No sequence homology of PTTH to any known protein has been found by database search, but three-dimensional modeling of the PTTH structure led to the conclusion that PTTH shares a common evolutionary origin with the vertebrate growth factor superfamily, including ***β***-nerve growth factor, transforming growth factor-***β***2, and platelet-derived growth factor-BB.29)

Two genes coding for prepro-PTTH-subunit were cloned from a *Bombyx* genomic DNA library using the PTTH cDNA as probe.30) These genes consist of five exons which are intervened by four introns. The 3’-terminal portion of the second exon, the third and fourth exons, and the 5’-terminal portion of the fifth exon encode the PTTH subunit (Fig. 8B). The PTTH gene exists as a single copy per *Bombyx* haploid genome, unlike the case of bombyxin where multiple gene copies are present.

## Studies using PTTH antibody

A monoclonal antibody recognizing *Bombyx* PTTH was raised against a synthetic peptide corresponding to the N-terminal 15-amino acid sequence of the PTTH subunit.31) This antibody immunostained two pairs of dorsolateral neurosecretory cells of *Bombyx* brain (Fig. 7C). The immunoreactive material was also detected in the axons of these neurosecretory cells which run across the brain midline to reach the contralateral corporus allatum (Fig. 7, Top and E), suggesting that PTTH is liberated into blood therefrom. The immunoreactive nerves in this case, however, were localized deep inside the allata (Fig. 7D), contrasting with the case of the bombyxin immunoreactive nerves. By *in situ* hybridization for PTTH mRNA using ^35^S-labeled cRNA as a probe, the same two dorsolateral neurosecretory cells of *Bombyx* brain were radiolabeled, confirming that PTTH is actually synthesized in these cells.26)

By a highly sensitive time-resolved fluoroimmunoassay using rabbit poly-clonal and mouse monoclonal antibodies against recombinant PTTH, developmental changes of PTTH titer in the *Bombyx* hemolymph have been monitored.32),33)

## Molecular biological approach to the inferrence of PTTH primary structure in other insects

No reliable sequence information from purified PTTH has been reported for any insect other than *Bombyx mori*, though a considerable degree of purification was achieved with PTTH of *Manduca sexta*34) and *Drosophila melanogaster*.35)

Molecular biological approach to infer the PTTH primary structure has accumulated recently, however. Based on the knowledge about the primary structure of *Bombyx*, the putative PTTH cDNA was cloned and the amino acid sequence of PTTH was deduced in such lepidopteran insects as *Samia cynthia ricini*,36) *Antheraea pernyi*,37) *Hyalophora cecropia*,38) *Manduca sexta*,39) *Helicoverpa zea*,40) and *Heliothis virescens*.41) The *Escherichia coli* recombinant PTTH of *A. pernyi* and *M. sexta* has been proved to be biologically active. Phylogenetic trees with respect to PTTH of these lepidopteran insects based on the amino acid sequence are presented.40),41)

The genes of *Anopheles gambiae*42) and *Drosophila melanogaster*43) have been assigned to the PTTH gene, based on the genome sequence of these dipteran insects. The putative PTTH peptides deduced from these genes show an extremely low homology with the lepidopteran PTTHs, except for seven Cys which are precisely conserved. It seems probable that the deipteran PTTH sequences assume the Cys-knot motif29) similar to that of *Bombyx* PTTH.
